# Treatment patterns, adverse events, healthcare resource use and costs among commercially insured patients with mantle cell lymphoma in the United States

**DOI:** 10.1002/cam4.2559

**Published:** 2019-10-08

**Authors:** Shaum M. Kabadi, Aimee Near, Keiko Wada, Chakkarin Burudpakdee

**Affiliations:** ^1^ AstraZeneca Gaithersburg MD USA; ^2^ IQVIA Falls Church VA USA

**Keywords:** adverse events, costs, healthcare resource use, Mantle cell lymphoma, real world, treatment patterns

## Abstract

**Introduction:**

There are limited data on treatment patterns, adverse events (AEs), and economic burden in younger, commercially insured patients treated for mantle cell lymphoma (MCL).

**Methods:**

Adults with ≥1 treatment for MCL between 1 November 2013‐31 December 2017 were identified from IQVIA Real‐World Data Adjudicated Claims‐US; index date was first treatment. Patients carried ≥1 MCL diagnosis, were newly treated, and were enrolled continuously for ≥12 months prior to and ≥30 days following index. Patients receiving the four most common MCL regimens were included. Measures included frequency of incident AEs, resource use, and costs overall and by number of AEs. Adjusted logistic regression and generalized linear modeling evaluated risk of hospitalization and all‐cause costs per patient per month (PPPM).

**Results:**

Two thousand five hundred and nine treated patients had a drug‐specific code and were classified to a specific treatment regimen. Of those patients, 1785 patients received at least one of the four most commonly used MCL regimens (R‐CHOP, rituximab monotherapy, B‐R, and ibrutinib) at some point over follow‐up (median 23 months). R‐CHOP was the most common regimen observed in the first line (26%), followed by rituximab monotherapy (19%), B‐R (15%), and ibrutinib (5%). The median age was 57 years; median Charlson Comorbidity Index was 0. Among patients receiving the four most common regimens, 63% of patients experienced ≥1 incident AE (R‐CHOP 77%, B‐R 58%, and ibrutinib 52%). An increasing number of incident AEs was associated with increased hospitalization risk (odds ratio = 2.4; 95% Confidence Interval [CI] 2.1‐2.7) and increased mean costs PPPM (cost ratio = 1.1; 95% CI 1.1‐1.2).

**Discussion:**

This is the largest study describing treatment patterns and clinical and economic impact of MCL treatment. The most common regimens were R‐CHOP, rituximab monotherapy, B‐R, and ibrutinib. The majority of treated patients experienced at least one incident AE, with hospitalization risk and all‐cause costs increasing as the number of AEs increased.

## Graphical abstract

This retrospective cohort study utilizing data from IQVIA Real‐World Data Adjudicated Claims‐US is the largest study describing treatment patterns and clinical and economic impact of MCL treatment. The most common regimens were R‐CHOP, rituximab monotherapy, B‐R, and ibrutinib. The majority of treated patients experienced at least one incident AE, with hospitalization risk and all‐cause costs increasing as the number of AEs increased.

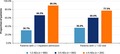

## 1 INTRODUCTION

Mantle cell lymphoma (MCL) is a rare, often aggressive B‐cell non‐Hodgkin's lymphoma (NHL), with a median age at diagnosis of 68.^1^ MCL accounts for 3% of all newly diagnosed NHL cases in the United States (US) with approximately 3300 new cases diagnosed in the US each year.^2^ Patient prognosis remains poor with a median overall survival of 4‐5 years.^3^

Current therapeutic approaches for MCL consider a variety of factors, including patient age and overall health status, treatment toxicity profiles, and patient and physician preferences,^4^ among other considerations. Treatments range from active observation, also called “wait‐and watch,” in the small proportion of patients with more indolent disease, to chemoimmunotherapy‐based regimens such as bendamustine and rituximab (B‐R) for patients not eligible for intensive therapy (eg, older individuals), to rituximab, cyclophosphamide, doxorubicin, vincristine, and prednisone (R‐CHOP) or dose‐intensified immunochemotherapeutic regimens with or without autologous stem cell transplantation in younger patients.^5^ Most patients with aggressive variants of MCL require treatment with immunochemotherapeutic regimens at the time of diagnosis; however, these treatments are not curative, with most patients eventually relapsing and requiring an alternative immunochemotherapy.^6^

As MCL treatments evolve and more intensive therapies are utilized to prolong survival, toxicity profiles for treatments should also be considered when developing the treatment plan. Younger patients who can tolerate intensive therapies may be more likely to experience acute or long‐term adverse effects, and the decision to undergo intensive treatment should carefully consider tradeoffs between efficacy and tolerability.^6^, ^7^, ^8^ In elderly patients, a standard first‐line therapy is B‐R, which is considered less toxic than dose‐intensive therapies and R‐CHOP.^7^, ^9^, ^10^, ^11^ Bruton tyrosine kinase (BTK) inhibitors, including ibrutinib (approved by the US Food and Drug Administration [FDA] in November 2013) and acalabrutinib (FDA approval in October 2017) have changed the treatment paradigm for patients with relapsed or refractory MCL.^4^, ^12^, ^13^ Other approved agents for relapsed or refractory MCL include lenalidomide (FDA approval in June 2013) and bortezomib which was first approved for previously treated MCL in 2006 and was expanded to include untreated MCL in 2014.^14^, ^15^ These novel agents carry unique adverse event profiles that should also be considered.

Furthermore, the cost of healthcare resource use (HRU) due to treatment toxicity can be substantial.^16^ One retrospective study of MCL patients initiating treatment between 2007 and 2011 reported a hospitalization rate of 45% during the 12 months before treatment began and 57% during the 12 months after treatment.^17^ The study also showed an increased number of emergency department (ED) and outpatient visits per patient in the 12 months after initiation of therapy versus the 12 months prior to treatment initiation (mean ED visits 0.5 before treatment vs. 0.8 after treatment; mean outpatient visits 31 before treatment vs. 63 after treatment).^17^ Another retrospective study reported that the mean monthly total healthcare cost increased from $1303 per patient during the 12 months before MCL diagnosis to $10964 after diagnosis; HRU and costs were higher among patients experiencing adverse events (AEs).^16^ Current data on treatment patterns, costs, and outcomes, particularly with the introduction of novel agent options, in the real‐world setting are limited.

To that end, measuring the occurrence of AEs and the economic burden of different MCL treatment regimens is of importance in an ever‐changing treatment landscape. Further evidence on the burden of novel and conventional therapies for MCL is crucial to patients and healthcare decision makers to guide treatment decisions. The purpose of this retrospective observational cohort study was to describe current treatment patterns, treatment‐related toxicity, and HRU and costs in a large, contemporary cohort of treated patients with MCL.

## 2 METHODS

### 2.1 Data source

This retrospective cohort study utilized patient data from the IQVIA Real‐World Data Adjudicated Claims—US (formerly known as PharMetrics Plus) database from 31 November 2012 to 31 January 2018. This database is comprised of adjudicated claims for more than 150 million unique commercially insured enrollees across the US, with data from 90% of US hospitals and 80% of all US doctors. Due to the broad reach of these data, records in the database are representative of the national, commercially insured population in terms of age and gender for individuals aged 65 and under. All data are HIPAA compliant to protect patient privacy.

### 2.2 Patient selection

**Figure 1:**
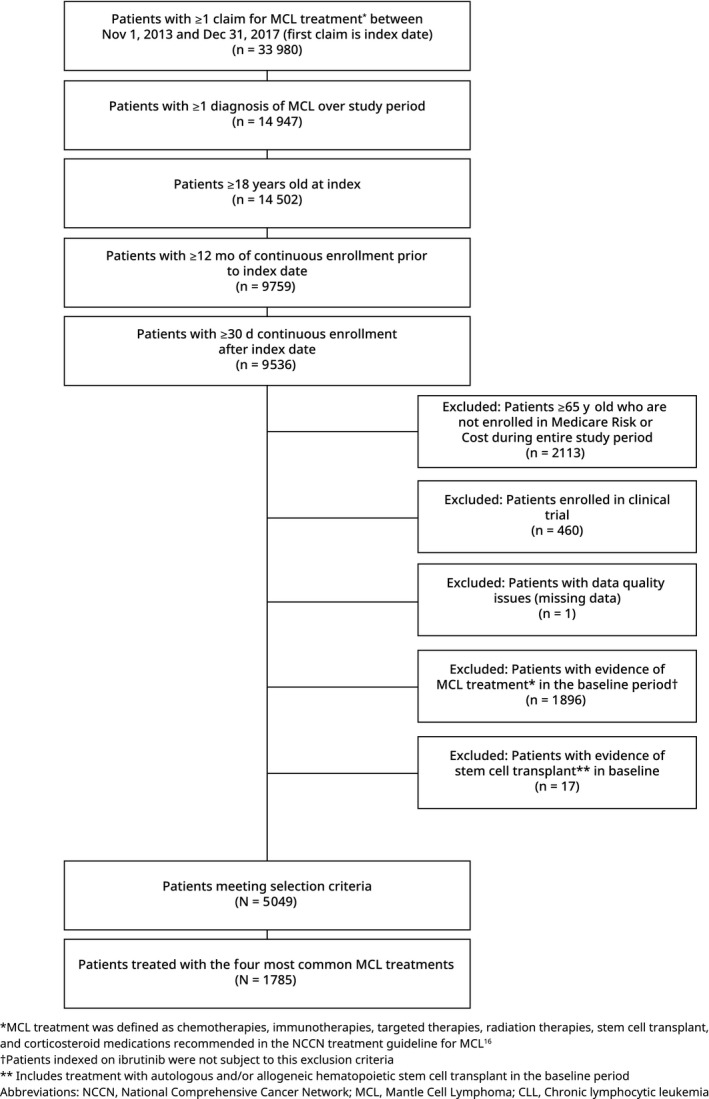
Patient attrition flowchart

Adult patients (age ≥ 18) who received at least one treatment for MCL (defined as treatments recommended by the National Comprehensive Cancer Network [NCCN guidelines]^7^ shown in Table S1) between 1 November 2013 and 31 December 2017 were identified. The first date of treatment was defined as the index date. Patients were required to have ≥ 1 diagnosis of MCL (International Classification of Diseases, Ninth Revision, Clinical Modification [ICD‐9‐CM] code 200.x or ICD‐10‐CM code C83.1x) during the study period and have ≥12 months of continuous enrollment prior to the index date (baseline period) and ≥30 days of continuous enrollment after the index date (variable follow‐up period). To ensure patients were newly treated, patients with evidence of MCL‐related treatment(s) during the baseline period were excluded. An exception to this criterion was made for patients indexed with ibrutinib, since ibrutinib is approved for the treatment of MCL in previously treated patients.^12^ The ibrutinib‐treated patients with prior MCL treatment (n = 16) were included in baseline measures but were excluded from analyses on AEs as well as follow‐up HRU and cost to minimize risk of misclassification of index line of therapy. Inclusion and exclusion criteria are described in Figure 1. After study selection was completed, patients who received one or more of the four most common treatment regimens at any point during follow‐up were included in the analysis.

### 2.3 Definitions of treatment episode and treatment regimen

The start of a treatment episode was defined as the first date of a MCL‐related systemic treatment. The combination of all agents used in the first 35 days of the beginning of a treatment episode comprised a treatment regimen; product‐specific drug and infusion administration codes (National Drug Codes [NDC] and Healthcare Common Procedure Coding System [HCPCS] codes) were used to identify treatments. Each treatment episode continued until a switch to a new regimen, modification of the starting regimen (addition and removal of rituximab were allowed, as this was expected in real‐world clinical practice), discontinuation of the regimen, end of study follow‐up, or end of continuous enrollment, whichever occurred first. When switching or modification of treatment occurred, the end date of a given treatment episode was defined as 1 day before the start date of the next treatment episode. Discontinuation was defined as a gap of ≥90 days in treatment. When discontinuation occurred, the end date was defined as 90 days after the last prescription was supplied (for oral medications) or 30 days after the last infusion was administered (for non‐oral medications). When systemic and oral treatments were used concomitantly, the end of the treatment episode was defined based on the oral medication supply. First, second, and third observed treatment episodes were identified and categorized as first‐, second‐, and third‐line therapies.

### 2.4 Study measures

#### 2.4.1 Baseline measures

Baseline demographic and clinical characteristics were measured during the 12‐month pre‐index period. Clinical characteristics included Charlson Comorbidity Index (CCI), atrial fibrillation (AF) risk status (based on risk factors present in the 12‐month baseline period, calculated using Chyou et al's method), 18 daily pill burden (defined as the total quantity of pills during the 30 days pre‐index, divided by 30 days, among patients with at least one oral prescription for MCL‐related treatments), and comorbidities (identified by ICD‐9‐CM, ICD‐10‐CM, and HCPCS codes). All baseline measures were reported for all patients.

#### 2.4.2 Postindex measures

Outcomes were collected during the variable follow‐up period (minimum 30 days), including number and frequency of patients treated with the four most common regimens by line of therapy, frequency of incident AEs of interest, and all‐cause and MCL‐related monthly HRU and costs per patient. Rituxumab monotherapy was only reported in the front line setting as distinguishing rituximab monotherapy from maintenance therapy in later lines of therapy was not possible. Patients were considered to have an incident AE (identified by ICD‐9‐CM, ICD‐10‐CM, and HCPCS codes) if they had ≥1 claim associated with an AE during a treatment episode with no evidence of that AE prior to the treatment initiation date. AEs of interest were selected from those previously observed in clinical trials,^19^, ^20^ package inserts,^7^ and expert clinical opinion of US‐based hematologists. MCL‐related HRU and costs were defined as the subset of all medical and inpatient claims with a diagnosis code for MCL at any position or claims (pharmacy and medical) for MCL‐related treatments. HRU and costs during follow‐up were reported separately for patients by the number of unique AEs (1‐2, 3‐5, and ≥6) and were categorized into hospitalizations, ED visits, outpatient visits, pharmacy, and other outpatient services (laboratory, ancillary, etc). Healthcare costs were based on the allowed amounts (negotiated rates between the plan and providers) and inflated to 2017 values using the Consumer Price Index.^21^

#### 2.4.3 Adjusted analyses

Generalized linear modeling with a log link and a gamma family distribution of the dependent variable and logistic regression were used to evaluate adjusted all‐cause healthcare cost per patient per month (PPPM) and the risk of hospitalization, respectively, among patients receiving the most common regimens in the first‐line setting. Model covariates included age at index (continuous), geographical region, insurance plan type, CCI score (continuous), baseline AF risk status, other baseline risk factors (evidence of infection, hypertension, anemia, fatigue/asthenia, hemorrhage/bleeding, and AF), treatment regimen, and number of incident AEs. All analyses were carried out using SAS version 9.2 software (SAS Inc., Cary, NC, USA).

## 3 RESULTS

### 3.1 Study population characteristics

Of the 5049 patients with MCL meeting the inclusion and exclusion criteria, 2509 patients had a drug‐specific code and were classified to a specific treatment regimen (eg rituximab monotherapy instead of unspecified biologic). Of these 2509 patients, 1785 patients received at least one of the four most commonly observed MCL treatment regimens at some point during follow‐up and were included in the study (Figure 1).

The mean ± standard deviation (SD) age was 55.8 ± 9.6 (median [interquartile range, IQR] 57.0 [52.0‐62.0]) years; 59.4% were male (Table 1). The geographic distribution of patients was diverse with most patients living in the South (35.2%) and Midwest (29.2%). The majority of patients were either commercially insured (57.5%) or self‐insured (33.6%). During the baseline period, the mean CCI score was 0.9 ± 1.4 (median 0 [0‐1]), 19.3% of patients were classified as high‐risk of AF, and the mean number of pills taken daily was 3.0 ± 3.7 (median 1.8 [0.2‐4.3]). The most common comorbid conditions were infection (46.6%), hypertension (39.7%), and anemia (32.3%). During the 12 months before MCL treatment initiation, patients incurred a mean all‐cause healthcare cost of $2946 ± $4160 PPPM (Table 1).

**Table 1:** Demographic and baseline clinical characteristics

Measures	All patients (n = 1785)**	
	N	%
Age at index, y		
Mean ± SD	55.8 ± 9.6	
Median (IQR)	57 (52, 62)	
18‐44	203	11.4
45‐54	411	23.0
55‐64	1088	61.0
65‐79	49	2.7
80 and older	34	1.9
Gender		
Male	1060	59.4
Female	725	40.6
Geographic region		
South	629	35.2
Midwest	522	29.2
Northeast	393	22.0
West	228	12.8
Unknown	13	0.7
Health plan type		
PPO	1395	78.2
HMO	236	13.2
POS	93	5.2
Other	45	2.5
Unknown	16	0.9
CCI Score		
Mean ± SD	0.9 ± 1.4	
Median (IQR)	0 (0, 1)	
Comorbid Conditions of Interest*		
Infection	831	46.6
Hypertension	708	39.7
Anemia	576	32.3
Fatigue/asthenia	401	22.5
Hemorrhage/bleeding	276	15.5
Thrombocytopenia	211	11.8
Edema	207	11.6
Nausea/vomiting	186	10.4
Neutropenia	128	7.2
Renal failure	127	7.1
Diarrhea	119	6.7
Pneumonia	110	6.2
Myalgia	99	5.6
Leukopenia	67	3.8
Atrial fibrillation	60	3.4
Hepatotoxicity	51	2.9
Atrial fibrillation risk status		
Low‐risk	1441	80.7
High‐risk	344	19.3
Daily Pill Burden, Pills		
Mean ± SD	3.0 ± 3.7	
Median (IQR)	1.8 (0.2, 4.3)	
Monthly all‐cause cost per patient during the 12 months baseline period, USD$		
Mean ± SD	$2946 ± $4160	
Median (IQR)	$1756 ($789, $3478)	

Abbreviations: CCI, Charlson Comorbidity Index; HMO, health maintenance organization; IQR, interquartile range; POS, point of service; PPO, preferred provider organization; SD, standard deviation.

* Conditions reported in ≥5% of all patients or included as the AEs of interest are reported in this table.

** The 1785 patients include all patients who received at least one of the four most commonly observed MCL treatment regimens at some point during follow‐up, regardless of line of therapy (ie patient could be indexed on another MCL treatment).

### 3.2 Treatment patterns and toxicity

**Figure 2:**
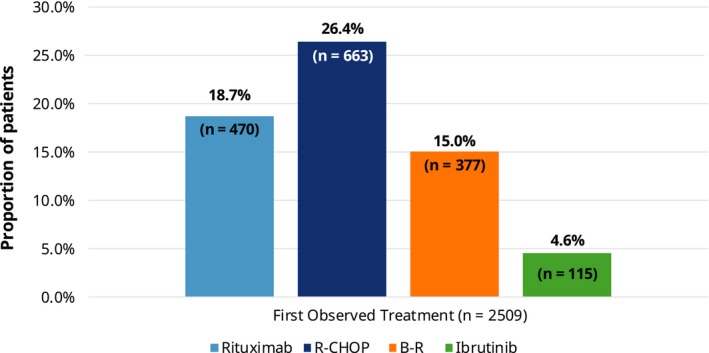
Patients treated with the four most common treatment regimens as the first observed therapy

The mean length of follow‐up after treatment initiation was 23.5 ± 14.4 months. The four most commonly observed MCL treatment regimens (with or without corticosteroids) across all observed lines of therapy were rituximab monotherapy (30.8% of all treated patients classified to specific regimen[s]), R‐CHOP (28.8%), B‐R (17.4%), and ibrutinib (7.9%). In the first‐line setting, there were 2509 patients who were classified to a specific treatment regimen, with R‐CHOP as the most common first observed regimen (26.4%), followed by rituximab monotherapy (18.7%), B‐R (15.0%), and ibrutinib (4.6%) (Figure 2). Among these patients, in the second‐ and third‐line settings, use of ibrutinib was most common (7.0% and 11.1%, respectively), followed by R‐CHOP (6.0% and 3.7%, respectively) and B‐R (5.3% and 4.3%, respectively). In addition, 7.0% of all treated patients, or 10.0% of patients receiving the four most common regimens, received autologous and/or allogeneic stem cell transplant at some point during the follow‐up.

Overall, 63.2% of patients newly treated with these regimens had at least one AE of interest (without regard to specific regimen or number of lines of therapy received). The mean number of AEs per patient over follow‐up was 1.1 ± 1.2 (median 1; IQR 0‐2). The most common AE, regardless of treatment regimen or line of therapy, was neutropenia (39.8%), followed by secondary malignancy (14.8%), anemia (10.6%), and infection (10.5%); 1.9% of patients experienced AF. The frequency of AEs during a given treatment varied by regimen, with the highest rate among R‐CHOP‐treated patients (77.0% had at least 1 AE; mean 1.3 ± 1.1 AEs per patient), followed by B‐R (58.1%; mean 0.9 ± 1.1 AEs per patient) and ibrutinib (51.9%; mean 1.1 ± 1.6 AEs per patient) (Table 2). Table 2 shows the number of AEs and the proportion of patients who experienced each AE of interest (not adjusted for age or CCI) by treatment regimen.

**Table 2:** Incident adverse events during treatment episode(s) by treatment regimen*

Incident adverse event	R‐CHOP (N = 721)		B‐R (N = 430)		Ibrutinib (N = 183)	
	N	%	N	%	N	%
Number of AEs†						
Mean ± SD	1.3 ± 1.1		0.9 ± 1.1		1.1 ± 1.6	
Median (IQR)	1 (1, 2)		1 (0, 1)		1 (0, 2)	
No AE	166	23.0	180	41.9	88	48.1
≥1 AE	555	77.0	250	58.1	95	51.9
Specific AE of interest						
Anemia	91	12.6	28	6.5	19	10.4
Arthralgia/Myalgia	10	1.4	10	2.3	8	4.4
Atrial fibrillation	11	1.5	5	1.2	10	5.5
Cerebrovascular	14	1.9	8	1.9	5	2.7
Diarrhea	24	3.3	16	3.7	15	8.2
Hemorrhage/Bleeding	24	3.3	5	1.2	22	12.0
Hepatotoxicity	6	0.8	2	0.5	2	1.1
Hypertension	23	3.2	18	4.2	17	9.3
Infection	65	9.0	39	9.1	21	11.5
Leukopenia	38	5.3	17	4.0	2	1.1
Myocardial infarction	7	1.0	2	0.5	4	2.2
Neutropenia	467	64.8	167	38.8	18	9.8
Renal failure	19	2.6	13	3.0	21	11.5
Secondary malignancy‡	84	11.7	51	11.9	21	11.5
Thrombocytopenia	34	4.7	25	5.8	23	12.6

Abbreviations: AE, adverse event; B‐R, bendamustine‐rituximab; IQR, interquartile range; R‐CHOP, cyclophosphamide/doxorubicin/vincristine/rituximab; SD, standard deviation

* Incident adverse events were measured among ibrutinib patients without evidence of prior treatment before index. Findings for patients receiving rituximab alone are not reported due to the lack of data to inform whether patients received rituximab monotherapy or rituximab maintenance therapy

^†^ For individual treatments, the AE must occur during the course of a given treatment (regardless of whether the treatment was the first/second/third). All AEs reported are incident AEs: Only AEs observed during treatment episode(s) but not observed prior to the start of treatment episode including baseline period are reported in this table

^‡^ Required at least two claims with diagnoses of the same cancer at least 7 d apart for the patient to be considered to have that secondary malignancy. ICD 9 code 202 and ICD 10 code C96 (“Other and unspecified malignant neoplasms of lymphoid, hematopoietic and related tissue”) were excluded from secondary malignancy.

### 3.3 HRU

**Figure 3:**
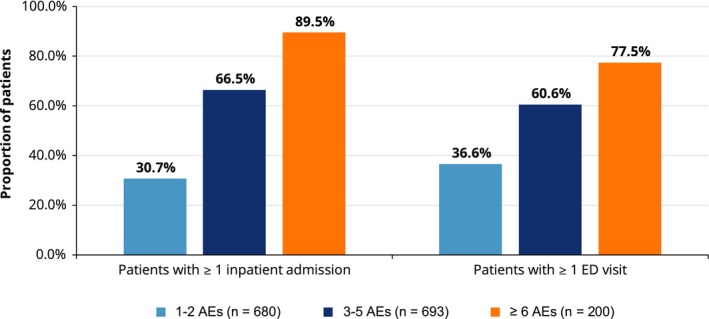
Healthcare resource use by number of incident adverse events

Among patients newly treated with the most common regimens, 49.7% had at least one hospitalization, with a mean of 0.1 ± 0.3 hospitalizations PPPM; the mean length of stay per inpatient admission was 3.7 ± 6.3 days (Table 3). Among patients with at least one AE, the proportion of patients requiring hospitalization increased as the number of unique incident AEs increased (ie from 30.7% of patients with 1‐2 AEs to 89.5% of patients with ≥6 AEs) (Figure 3; Table 3). The mean length of stay per inpatient admission also increased from 2.1 ± 5.8 days among patients with 1‐2 AEs to 7.7 ± 5.8 days among patients with ≥6 AEs. Furthermore, 49.3% of all patients had at least one ED visit, with a mean of 0.1 ± 0.2 visits PPPM (Table 3). The proportion of patients with at least one ED visit also increased as the number AEs increased, from 36.6% of patients with 1‐2 AEs to 77.5% of patients with ≥6 AEs. Almost all patients had at least one office visit (99.7%) and other outpatient services such as laboratory or ancillary (99.9%), with a mean of 2.0 ± 1.5 office visits and 13.5 ± 11.4 claims PPPM for other outpatient services, respectively. Mean all‐cause pharmacy use was 6.6 ± 5.2 prescriptions/injection administrations PPPM. Similar to hospitalizations and ED visits, increased use of office visits, pharmacy and other outpatient services was observed as the number of AEs increased (Table 3). Trends for MCL‐related HRU were similar to all‐cause findings described and are shown in Table 3.

**Table 3:** All‐cause and MCL‐related monthly healthcare utilization per patient among all patients by number of unique incident AEs experienced

Monthly healthcare utilization per patient*	All‐cause utilization								MCL‐related utilization§							
	All pts‡		1‐2 AEs		3‐5 AE		≥6 AEs		All pts‡		1‐2 AEs		3‐5 AE		≥6 AEs	
	(N = 1768)		(N = 680)		(N = 693)		(N = 200)		(N = 1768)		(N = 680)		(N = 693)		(N = 200)	
	N	%	N	%	N	%	N	%	N	%	N	%	N	%	N	%
Hospitalization																
Pts w/ ≥1 admission	879	49.7	209	30.7	461	66.5	179	89.5	497	28.1	99	14.6	262	37.8	122	61.0
Mean, SD	0.1 ± 0.3		0.1 ± 0.2		0.2 ± 0.3		0.3 ± 0.4		0.1 ± 0.2		0.0 ± 0.1		0.1 ± 0.2		0.1 ± 0.2	
Median	0.0		0.0		0.0		0.2		0.0		0.0		0.0		0.0	
LOS per admission (days)																
Mean, SD	3.7 ± 6.3		2.1 ± 5.8		4.9 ± 6.6		7.7 ± 5.8		2.6 ± 7.2		1.2 ± 5.5		3.6 ± 8.4		5.7 ± 7.4	
Median	0.0		0.0		4.0		6.9		0.0		0.0		0.0		4.3	
ED visit																
Pts w/ ≥1 visit	872	49.3	249	36.6	420	60.6	155	77.5	128	7.2	29	4.3	63	9.1	34	17.0
Mean, SD	0.1 ± 0.2		0.0 ± 0.1		0.1 ± 0.2		0.2 ± 0.2		0.0 ± 0.0		0.0 ± 0.0		0.0 ± 0.0		0.0 ± 0.1	
Median	0.0		0.0		0.0		0.1		0.0		0.0		0.0		0.0	
Office visit																
Pts w/ ≥1 visit	1763	99.7	678	99.7	693	100.0	200	100.0	1207	68.3	444	65.3	506	73.0	153	76.5
Mean, SD	2.0 ± 1.5		1.8 ± 1.4		2.1 ± 1.6		2.7 ± 1.7		0.4 ± 0.8		0.4 ± 0.7		0.5 ± 0.8		0.6 ± 0.9	
Median	1.6		1.4		1.6		2.2		0.1		0.1		0.1		0.2	
Pharmacy																
Pts w/ ≥1 prescription	1768	100.0	680	100.0	693	100.0	200	100.0	1768	100.0	680	100.0	693	100.0	200	100.0
Mean, SD	6.6 ± 5.2		6.7 ± 5.9		6.3 ± 4.6		7.7 ± 5.0		2.3 ± 2.6		2.5 ± 2.8		2.1 ± 2.4		2.0 ± 1.8	
Median	5.1		5.0		5.0		6.4		1.4		1.4		1.3		1.3	
Other services†																
Pts w/ ≥1 claim	1766	99.9	680	100.0	693	100.0	200	100.0	1714	96.9	663	97.5	676	97.5	197	98.5
Mean, SD	13.5 ± 11.4		12.2 ± 10.8		14.0 ± 11.9		18.3 ± 11.8		4.6 ± 6.7		4.1 ± 5.7		4.7 ± 7.3		5.5 ± 8.0	
Median	9.9		8.5		10.0		14.8		2.1		2.1		2.1		2.4	

Abbreviations: AE, adverse event; ED, Emergency department; LOS, length of stay; MCL, Mantle cell lymphoma; Pts, patients; SD, standard deviation; w/, with.

* For patients receiving ibrutinib, HRU and costs were measured among ibrutinib patients without evidence of prior treatment before index.

^†^ Other services include laboratory and pathology, radiology, surgery, ancillary and all other outpatient services.

^‡^ All patients include patients with no new AEs during treatment and are patients who received at least one of the four most commonly observed MCL treatment regimens at some point during follow‐up, regardless of line of therapy (ie patient could be indexed on another MCL treatment).

^§^ MCL‐related utilization was defined as medical and inpatient claims with a diagnosis code for MCL at any positions or pharmacy and medical claims for MCL‐related treatments

### 3.4 Healthcare costs

**Figure 4:**
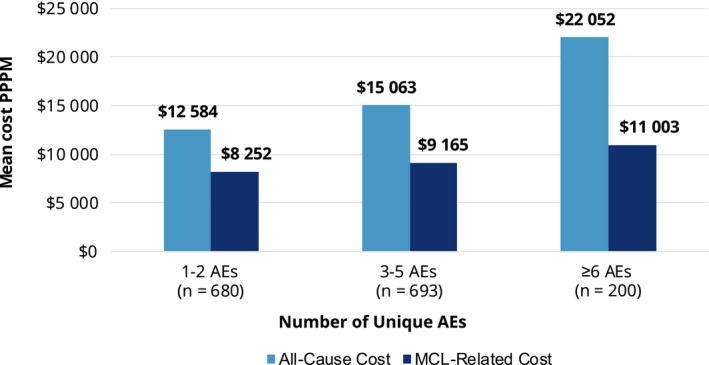
All‐cause and MCL‐related costs by number of incident adverse events

The mean total all‐cause healthcare cost over the follow‐up was $14 786 ± $16 482 PPPM. Total MCL‐related costs (mean $9267 ± $12 309 PPPM) accounted for 62.7% of the total all‐cause costs (Table 4). The largest contributor to total all‐cause and total MCL‐specific costs was medication costs (mean $7094 ± $6736 for all‐cause and $5645 ± $5780 for MCL‐related). Inpatient cost was the second largest contributor for both all‐cause and MCL‐specific costs (Table 4). Among patients with at least one incident AE, all‐cause cost PPPM increased as the number of AEs increased, from $12 584 for patients with 1‐2 AEs to $22 052 for patients with ≥6 AEs; This trend was consistent for MCL‐specific costs (Table 4, Figure 4). Mean all‐cause and MCL‐related costs PPPM for all resource categories except for pharmacy and other outpatient services increased as the number of AEs increased (Table 4).

**Table 4:** All‐cause and MCL‐related monthly healthcare costs per patient by number of unique AEs experienced

Monthly healthcare cost per patient (USD)*	All‐cause cost				MCL‐related cost§			
	All pts‡ (N = 1768)	1‐2 AEs (N = 680)	3‐5 AE (N = 693)	≥6 AEs (N = 200)	All pts‡ (N = 1768)	1‐2 AEs (N = 680)	3‐5 AE (N = 693)	≥6 AEs (N = 200)
Total cost								
Mean	$14 786	$12 584	$15 063	$22 052	$9267	$8252	$9165	$11 003
SD	$16 482	$12 717	$17 751	$18 727	$12 309	$8667	$13 247	$13 474
Median	$9637	$8416	$9147	$16 022	$5457	$5024	$5028	$6473
Hospitalization								
Mean	$4489	$2046	$5532	$11 411	$2399	$962	$3305	$5159
SD	$12 341	$6824	$13 973	$14 214	$9217	$4432	$10 631	$8600
Median	$0	$0	$937	$6644	$0	$0	$0	$805
ED visit								
Mean	$87	$43	$115	$173	$8	$3	$12	$20
SD	$334	$122	$481	$307	$72	$33	$88	$118
Median	$0	$0	$17	$60	$0	$0	$0	$0
Office visit								
Mean	$280	$267	$275	$402	$63	$58	$63	$85
SD	$491	$700	$217	$482	$155	$201	$116	$136
Median	$195	$181	$208	$294	$15	$13	$17	$26
Pharmacy								
Mean	$7094	$7623	$6235	$6121	$5645	$6130	$4700	$4227
SD	$6736	$7552	$5684	$5027	$5780	$6159	$4600	$4641
Median	$4955	$5297	$4622	$4720	$3774	$4010	$3160	$2945
Other services†								
Mean	$2835	$2606	$2906	$3944	$1152	$1099	$1085	$1512
SD	$3760	$3580	$3338	$5526	$2785	$2432	$2207	$4860
Median	$1714	$1525	$1820	$2545	$407	$401	$384	$438

Abbreviations: AE, adverse event; ED, Emergency department; Pts, patients; SD, standard deviation; w/, with.

* For patients receiving ibrutinib, HRU and costs were measured among ibrutinib patients without evidence of prior treatment before index.

^†^ Other services include laboratory and pathology, radiology, surgery, ancillary and all other outpatient services

^‡^ All patients include patients with no new AEs during treatment and are patients who received at least one of the four most commonly observed MCL treatment regimens at some point during follow‐up, regardless of line of therapy (ie patient could be indexed on another MCL treatment).

^§^ MCL‐related utilization was defined as medical and inpatient claims with a diagnosis code for MCL at any positions or pharmacy and medical claims for MCL‐related treatments.

### 3.5 Adjusted analyses among patients treated in the first‐line setting

Among patients newly receiving the four most common regimens in the first‐line setting (n = 1614), a logistic regression model, controlling for demographic and clinical characteristics and regimen, confirmed the increased odds of hospitalization as the number of incident AEs increased (odds ratio [OR] = 2.4; 95% Confidence Interval [CI] 2.1‐2.7; *P*<.0001; Table S2). When the impact of each AE of interest on hospitalization was evaluated, patients with incident AF had the highest odds of hospitalization (OR = 5.8; 95% CI 2.2‐15.6; *P *= .0005), followed by patients with incident anemia (OR = 5.3; 95% CI 3.6‐7.9; *P *< .0001) and incident thrombocytopenia (OR = 5.0; 95% CI 3.0‐8.1; *P *< .0001) (Table S4).

A generalized linear model, controlling for demographic and clinical characteristics and regimen, showed a trend toward higher total all‐cause cost PPPM as the number of incident AEs increased (cost ratio [CR] = 1.1, 95% CI 1.1‐1.2, *P *< .0001; Table S3). When the impact of each AE of interest on total all‐cause cost PPPM was evaluated, patients with incident thrombocytopenia incurred the highest costs (CR = 1.6; 95% CI 1.4‐2.0; *P *< .0001) (Table S5).

## 4 DISCUSSION

This study describes the most common MCL treatments and associated toxicities, HRU and costs in the largest sample of treated MCL patients to date. Patients in our study were younger than the general MCL population in the US (median age was 57 years in our study compared to 68 years in the general MCL population^1^), reflecting the commercially insured population in the database we used. At the time of treatment, patients had low comorbidity burden (median CCI score 0, mean 0.9), which was lower than the mean CCI score of 2, reported in another retrospective claims analysis of older patients newly diagnosed with MCL.^16^ Approximately 59% of patients were male in our study, similar to the reported MCL male‐to‐female ratio of 3:1.^1^, ^22^

Our study included patients treated for MCL in recent years and provides data on the rates of use and associated toxicity of the most commonly observed MCL regimens. While we did not capture less frequently used treatment regimens, this study provides insight into current MCL treatments utilized among younger, commercially insured MCL patients. The most common treatments observed were rituximab alone, R‐CHOP, B‐R, and ibrutinib. This is consistent with a recent retrospective claims analysis that evaluated treatment patterns among newly diagnosed MCL patients, except that our study found a lower proportion of patients treated with B‐R, likely due to our younger study population.^16^

Further, this study highlights the clinical and economic burden among newly treated MCL patients. The majority of patients studied experienced at least one incident AE during their treatment (63%). Among the three most common MCL treatment regimens, the frequency of incident AEs was highest among patients treated with R‐CHOP (77%), followed by B‐R (58%) and ibrutinib (52%). We did not report the frequency for patients receiving rituximab alone given the possibility of some of these patients receiving rituximab as maintenance therapy, suggesting lower tumor burden compared to patients receiving the other three treatments. As suggested by a prior study,^23^ the rate of AEs in clinical practice is higher than rates reported in clinical trials.^10^, ^11^, ^24^, ^25^ The most commonly observed incident AE in our study was neutropenia, followed by secondary malignancy, anemia, and infection; findings generally consistent with previous real‐world studies and clinical trials for each treatment regimen.^4^, ^26^ For ibrutinib, the phase 2 trial in relapsed and refractory MCL upon which FDA approval of the agent was granted, reported that the most common AEs were diarrhea (54%), fatigue (50%), and nausea (33%) while we found that thrombocytopenia (13%) was the most common AE among ibrutinib‐treated patients. We additionally found that hemorrhage/bleeding (12%), infection (12%), renal failure (12%), and secondary malignancy (12%) were reported for ibrutinib‐treated patients.^19^ This discrepancy is not surprising as our study leverages claims data, which may underreport AEs not requiring treatment or office visits.

Overall, we found that the proportion of patients utilizing each healthcare resource increased as the number of incident AEs increased. In our study, 50% of patients were hospitalized during the follow‐up period. Although our follow‐up was longer (median 23 months) than a prior study, this is consistent with another retrospective claims study of MCL patients (majority were newly treated with R‐CHOP, B‐R, or rituximab monotherapy) which reported a hospitalization rate of 57% over the 12 months following treatment initiation.^17^ Our study also showed that the mean length of stay among patients who had 1‐2 AEs was 2 days compared to 8 days among patients with ≥ 6 AEs. One recent claims analysis of older MCL patients reported 4 days and 5 days for patients with 1‐2 and ≥ 6 AEs, respectively.^16^ Our findings show a consistent trend observed in prior studies, though slight differences in numbers are likely due to our younger, healthier population managed with intensive treatments.

Our findings of HRU by the number of AEs suggest an association between treatment toxicity, HRU, and cost, thus highlighting the benefit of treatments with more favorable toxicity profiles. For example, both the hospitalization rate and mean length of stay among patients experiencing 1‐2 AEs were less than half of those of patients experiencing 3‐5 AEs. This suggests that decreasing toxicity may reduce HRU burden among MCL patients. Adjusted analyses further confirmed the trend towards higher risk of hospitalization as the number of AEs increased. Our results are aligned with a claims‐based study reporting that increased AEs resulted in increased HRU, including hospitalization and ED visits.^16^

Furthermore, our study quantified the current economic impact among newly treated MCL patients. Among patients treated with the four most commonly observed regimens, the mean total healthcare cost after initial treatment ($14786 PPPM) was five times higher than it was in the 12 months before treatment initiation (mean $2946 PPPM). This cost after initial treatment was higher but comparable to the mean total cost reported in a similar claims‐based analysis.^16^ The higher costs in our study may be explained by our younger population treated with more intensive regimens, resulting in more AEs, HRU, and associated costs. Pharmacy costs accounted for nearly two thirds of total cost, while all‐cause inpatient and other outpatient services such as laboratory or ancillary services were the next most significant contributors to cost. The unadjusted costs rose as the number of AEs increased, and this finding was supported by the adjusted cost model and consistent with previous studies.^16^, ^27^ The adjusted analyses further suggest MCL patients experiencing thrombocytopenia incurred the highest monthly healthcare costs and an increased rate of hospitalization compared to those without thrombocytopenia, perhaps given increased bleeding events.

This study is subject to limitations common to retrospective database analyses including possible billing and coding errors, and the fact that the data were not collected for research purposes. Our study leveraged administrative claims data. Due to the lack of clinical information in the claims database (eg, prognostic factors including lactate dehydrogenase, white blood cell count), we were unable to assess tumor burden and other factors which influence treatment choice. Given this limitation, it is possible that patients in our study receiving rituximab as the second or third observed therapy could be maintenance therapy patients. To account for this limitation, we did not compare study outcomes between patients receiving rituximab alone and patients receiving other regimens. In addition, the high frequency of first‐line rituximab monotherapy observed in our study should be interpreted with caution as it does not align with conventional clinical practice. This unexpected observation is likely a reflection of one or several of the following limitations of this claims‐based analysis. First, while we excluded patients with evidence of clinical trial enrollment in this study, the code for trial participation is underreported in claims, and it is possible that patients appearing to receive rituximab monotherapy were in fact enrolled in trials, receiving study drug(s) and rituximab as part of the trial. As rituximab is commercially available, it is possible that rituximab was submitted to insurance while other study drug(s) were provided free by the trial. Second, stem cell transplant is also underreported in claims data, and although prior stem cell transplant was an exclusion criterion, it is possible that these rituximab patients had prior transplant that we cannot observe in the database. Finally, prior MCL therapy given before the available look‐back period is a possibility among study patients and is an inherent limitation of all database studies with a defined data window. With that said, one retrospective cohort study in a community setting reported 18% of first‐line treatment for MCL was rituximab monotherapy,^28^ which is consistent with the frequency observed in our study (19%); however, this study has limitations similar to our study that rituximab monotherapy as a line of therapy cannot be distinguished from maintenance rituximab based on electrical medical record data.

Furthermore, clinical information to assess severity and additional details for the observed AEs were also not available in claims data. In order to explore the burden of AEs associated with treatments, we measured and compared HRU and costs among patients experiencing 1‐2, 3‐5, and ≥6 incident AEs identified using diagnosis codes. A causal relationship between AEs, HRU, and cost cannot be established without additional clinical information. Moreover, the duration of follow‐up was limited in some patients, resulting in a small number of patients receiving second‐ and third‐line therapy. Therefore, longer follow‐up in a larger sample of patients would likely provide a more comprehensive picture of differences among treatment regimens over time. Lastly, as previously mentioned, use of stem cell transplant was underreported in this study, limiting our ability to determine the frequency of stem cell transplant and costs associated with this procedure. We excluded the few patients with evidence of autologous and/or allogeneic stem cell transplant during the baseline period from the study because their clinical profile and outcomes are expected to be different from patients who did not have stem cell transplant. Despite these limitations, the volume and comprehensiveness of the IQVIA Real‐World Data Adjudicated Claims–US database provides the ability to observe treatments, events, HRU, and costs in all settings of care longitudinally.

In this study of patients treated in clinical practice, the most common regimens observed among MCL patients were rituximab, R‐CHOP, B‐R, and ibrutinib. More than half of newly treated patients experienced at least one incident AE. HRU and costs consistently increased as the number of AEs increased, suggesting an association between greater treatment toxicity and higher HRU. Adjusted models confirmed the trend of the healthcare costs and odds of hospitalization increasing as the number of AEs increase. This study highlights the clinical and economic burden associated with current, commonly used MCL treatments. As novel therapies are increasingly available and used, further research examining clinical and economic outcomes will inform prescribers and patients as they aim to understand the risks, benefits, and value of novel agents.
