# Supplementary material for: Treatment patterns, adverse events, healthcare resource use and costs among commercially insured patients with mantle cell lymphoma in the United States
Source: Cancer Med. 2019 Oct 8;8(17):7174–85. doi: 10.1002/cam4.2559 (PMC6885896; doi:10.1002/cam4.2559)
Supplement: Supplementary file 1 [file CAM4-8-7174-s001.docx]

**Supplemental Table 1. List of National Comprehensive Cancer Network (NCCN) Guideline-Recommended Treatments for Mantle Cell Lymphoma**

| **Chemotherapy** |
| --- |
| Bendamustine |
| Carboplatin |
| Carmustine |
| Cyclophosphamide |
| Cytarabine |
| Doxorubicin |
| Etoposide |
| Fludarabine |
| Ifosfamide |
| Methotrexate |
| Mitoxantrone |
| Pentostatin |
| Procarbazine |
| Vincristine |
| Chemotherapy administration/unspecified |
| **Biologic therapy/Immunomodulators** |
| Lenalidomide |
| Rituximab |
| Unclassified biologics |
| **Targeted therapy** |
| Bortezomib |
| Ibrutinib |
| **Corticosteroids** |
| Dexamethasone |
| Prednisone |

**Supplemental Table 2. Multivariable Logistic Regression Model Assessing Association Between Number of Incident Adverse Events and Inpatient Admission During the First Observed Treatment Episode**

| **Outcome:  Inpatient Admission (Yes/No)** | **Among Patients Receiving First Observed Treatment**  **n = 1,614** | | | | | | |
| --- | --- | --- | --- | --- | --- | --- | --- |
|  | **Parameter Estimate** | **Standard Error** | **Chi-Square** | **P-value** | **Odds Ratio** | **95% CI** | |
| **Number of Incident Adverse Events*** |  |  |  |  |  |  |  |
| **Continuous** | 0.88 | 0.06 | 194.64 | <.0001 | 2.42 | 2.14 | 2.74 |
| **Age at Index** |  |  |  |  |  |  |  |
| **Continuous** | -0.01 | 0.01 | 2.18 | 0.14 | 0.99 | 0.98 | 1.00 |
| **Region (ref = South)** |  |  |  |  |  |  |  |
| **Northeast** | 0.66 | 0.18 | 12.80 | 0.00 | 1.93 | 1.35 | 2.77 |
| **Midwest** | 0.54 | 0.17 | 10.22 | 0.00 | 1.71 | 1.23 | 2.38 |
| **West** | 0.43 | 0.23 | 3.43 | 0.06 | 1.54 | 0.98 | 2.43 |
| **Unknown** | 1.32 | 0.64 | 4.18 | 0.04 | 3.73 | 1.06 | 13.17 |
| **Plan Type (ref = PPO)** |  |  |  |  |  |  |  |
| **HMO** | 0.19 | 0.20 | 0.94 | 0.33 | 1.21 | 0.82 | 1.78 |
| **POS** | 0.25 | 0.28 | 0.81 | 0.37 | 1.29 | 0.74 | 2.24 |
| **Other and Unknown** | 0.08 | 0.34 | 0.06 | 0.80 | 1.09 | 0.56 | 2.11 |
| **CCI Score** |  |  |  |  |  |  |  |
| **Continuous** | 0.12 | 0.05 | 4.62 | 0.03 | 1.12 | 1.01 | 1.25 |
| **AF Risk Status at Baseline (ref = Low-risk)** |  |  |  |  |  |  |  |
| **High-risk** | -0.14 | 0.22 | 0.42 | 0.52 | 0.87 | 0.56 | 1.34 |
| **Other Baseline Risk Factors (Yes/No)** |  |  |  |  |  |  |  |
| **Infection** | 0.36 | 0.14 | 7.15 | 0.01 | 1.44 | 1.10 | 1.88 |
| **Hypertension** | 0.17 | 0.15 | 1.18 | 0.28 | 1.18 | 0.88 | 1.60 |
| **Anemia** | 0.49 | 0.14 | 11.39 | 0.00 | 1.63 | 1.23 | 2.17 |
| **Fatigue/asthenia** | 0.18 | 0.16 | 1.27 | 0.26 | 1.19 | 0.88 | 1.62 |
| **Hemorrhage/bleeding** | 0.07 | 0.18 | 0.14 | 0.71 | 1.07 | 0.76 | 1.51 |
| **Atrial fibrillation** | 0.68 | 0.34 | 3.96 | 0.05 | 1.98 | 1.01 | 3.87 |
| **Treatment Regimen (ref = Rituximab)** |  |  |  |  |  |  |  |
| **R-CHOP** | -0.23 | 0.16 | 2.08 | 0.15 | 0.79 | 0.58 | 1.09 |
| **B-R** | -1.00 | 0.21 | 23.27 | <.0001 | 0.37 | 0.24 | 0.55 |
| **Ibrutinib** | 0.18 | 0.27 | 0.46 | 0.50 | 1.20 | 0.71 | 2.04 |
| *Occurred During a Given Treatment Episode, selected based on conditions observed in≥5% of patients treated with top 4 regimens and clinical relevance  Abbreviations: AF, atrial fibrillation; B-R, bendamustine-rituximab; CCI, Charlson comorbidity index; CI, confidence interval; HMO, health maintenance organization; POS, point of service; PPO, preferred provider organization; R-CHOP, cyclophosphamide/doxorubicin/vincristine/rituximab | | | | | | | |

**Supplemental Table 3. Multivariable Generalized Linear Model Assessing Association Between Number of Incident Adverse Events and Monthly All-Cause Health Care Costs per Patient During the Entire Study Follow-up**

| **Outcome:  All-Cause Healthcare Costs PPPM** | **Patients Receiving First Observed Treatment for the Four Most Common Regimens** | | | | | | | | |
| --- | --- | --- | --- | --- | --- | --- | --- | --- | --- |
|  | **n = 1,614** | | | | | | | | |
|  | **Parameter Estimate** | **Standard Error** | **Chi-Square** | **p-value** | **95% CI** | | **Cost Ratio** | **95% CI** | |
| **Number of Incident Adverse Events** |  |  |  |  |  |  |  |  |  |
| **Continuous** | 0.13 | 0.02 | 51.47 | <.0001 | 0.09 | 0.17 | 1.14 | 1.10 | 1.18 |
| **Age at Index** |  |  |  |  |  |  |  |  |  |
| **Continuous** | -0.01 | 0.00 | 12.91 | 0.00 | -0.01 | 0.00 | 0.99 | 0.99 | 1.00 |
| **Region (ref = South)** |  |  |  |  |  |  |  |  |  |
| **Northeast** | 0.13 | 0.06 | 4.69 | 0.03 | 0.01 | 0.25 | 1.14 | 1.01 | 1.28 |
| **Midwest** | 0.15 | 0.05 | 7.76 | 0.01 | 0.04 | 0.25 | 1.16 | 1.04 | 1.29 |
| **West** | 0.15 | 0.07 | 4.09 | 0.04 | 0.00 | 0.29 | 1.16 | 1.00 | 1.34 |
| **Unknown** | -0.32 | 0.24 | 1.68 | 0.19 | -0.79 | 0.16 | 0.73 | 0.45 | 1.17 |
| **Plan Type (ref = PPO)** |  |  |  |  |  |  |  |  |  |
| **HMO** | -0.21 | 0.07 | 10.31 | 0.00 | -0.34 | -0.08 | 0.81 | 0.71 | 0.92 |
| **POS** | 0.14 | 0.09 | 2.21 | 0.14 | -0.04 | 0.32 | 1.15 | 0.96 | 1.37 |
| **Other and Unknown** | 0.24 | 0.12 | 4.12 | 0.04 | 0.01 | 0.46 | 1.27 | 1.01 | 1.59 |
| **CCI Score** |  |  |  |  |  |  |  |  |  |
| **Continuous** | 0.06 | 0.02 | 10.98 | 0.00 | 0.03 | 0.10 | 1.07 | 1.03 | 1.11 |
| **AF Risk Status at Baseline (ref = Low-risk)** |  |  |  |  |  |  |  |  |  |
| **High-risk** | -0.14 | 0.07 | 3.82 | 0.05 | -0.29 | 0.00 | 0.87 | 0.75 | 1.00 |
| **Other Baseline Risk Factors (Yes/No)** |  |  |  |  |  |  |  |  |  |
| **Infection** | -0.03 | 0.04 | 0.61 | 0.43 | -0.12 | 0.05 | 0.97 | 0.89 | 1.05 |
| **Hypertension** | 0.15 | 0.05 | 9.10 | 0.00 | 0.05 | 0.25 | 1.16 | 1.05 | 1.28 |
| **Anemia** | 0.19 | 0.05 | 15.95 | <.0001 | 0.10 | 0.29 | 1.21 | 1.10 | 1.34 |
| **Fatigue/asthenia** | 0.13 | 0.05 | 6.53 | 0.01 | 0.03 | 0.24 | 1.14 | 1.03 | 1.27 |
| **Hemorrhage/bleeding** | -0.03 | 0.06 | 0.29 | 0.59 | -0.15 | 0.09 | 0.97 | 0.86 | 1.09 |
| **Atrial fibrillation** | 0.37 | 0.12 | 8.62 | 0.00 | 0.12 | 0.61 | 1.44 | 1.13 | 1.84 |
| **Treatment Regimen (ref = Rituximab)** |  |  |  |  |  |  |  |  |  |
| **R-CHOP** | 0.01 | 0.05 | 0.07 | 0.80 | -0.09 | 0.11 | 1.01 | 0.92 | 1.12 |
| **B-R** | 0.09 | 0.06 | 2.43 | 0.12 | -0.02 | 0.21 | 1.10 | 0.98 | 1.23 |
| **Ibrutinib** | 0.37 | 0.09 | 17.15 | <.0001 | 0.20 | 0.55 | 1.45 | 1.22 | 1.74 |
| Abbreviations: AF, atrial fibrillation; B-R, bendamustine-rituximab; CCI, Charlson comorbidity index; CI, confidence interval; HMO, health maintenance organization; POS, point of service; PPO, preferred provider organization; PPPM, per patient per month; R-CHOP, cyclophosphamide/doxorubicin/vincristine/rituximab | | | | | | | | | |

**Supplemental Table 4. Multivariable Logistic Regression Model Assessing Association Between Incident Adverse Events of Interest and Inpatient Admission During the First Observed Treatment Episode**

| **Outcome:  Inpatient Admission (Yes/No)** | **Patients Receiving First Observed Treatment for the Four Most Common Regimens** | | | | | | |
| --- | --- | --- | --- | --- | --- | --- | --- |
|  | **n = 1,614** | | | | | | |
|  | **Parameter Estimate** | **Standard Error** | **Chi-Square** | **P-value** | **Odds Ratio** | **95% CI** | |
| **Incident Adverse Event (Yes/No)*** |  |  |  |  |  |  |  |
| **Neutropenia** | 0.41 | 0.16 | 6.74 | 0.01 | 1.51 | 1.11 | 2.06 |
| **Secondary malignancy** | 0.11 | 0.20 | 0.33 | 0.57 | 1.12 | 0.76 | 1.65 |
| **Anemia** | 1.67 | 0.20 | 70.23 | <.0001 | 5.32 | 3.60 | 7.86 |
| **Infection** | 0.73 | 0.22 | 11.22 | 0.00 | 2.08 | 1.36 | 3.20 |
| **Thrombocytopenia** | 1.60 | 0.25 | 40.74 | <.0001 | 4.95 | 3.03 | 8.09 |
| **Atrial fibrillation** | 1.76 | 0.50 | 12.18 | 0.00 | 5.82 | 2.16 | 15.63 |
| **Hemorrhage/bleeding** | 1.45 | 0.32 | 20.88 | <.0001 | 4.28 | 2.29 | 7.98 |
| **Age at Index** |  |  |  |  |  |  |  |
| **Continuous** | -0.01 | 0.01 | 1.32 | 0.25 | 0.99 | 0.98 | 1.01 |
| **Region (ref = South)** |  |  |  |  |  |  |  |
| **Northeast** | 0.61 | 0.18 | 11.24 | 0.00 | 1.84 | 1.29 | 2.63 |
| **Midwest** | 0.49 | 0.17 | 8.44 | 0.00 | 1.63 | 1.17 | 2.26 |
| **West** | 0.36 | 0.23 | 2.34 | 0.13 | 1.43 | 0.91 | 2.26 |
| **Unknown** | 1.37 | 0.64 | 4.66 | 0.03 | 3.95 | 1.13 | 13.77 |
| **Plan Type (ref = PPO)** |  |  |  |  |  |  |  |
| **HMO** | 0.10 | 0.20 | 0.27 | 0.60 | 1.11 | 0.75 | 1.63 |
| **POS** | 0.23 | 0.28 | 0.66 | 0.42 | 1.26 | 0.73 | 2.18 |
| **Other and Unknown** | 0.20 | 0.33 | 0.38 | 0.54 | 1.23 | 0.64 | 2.34 |
| **CCI Score** |  |  |  |  |  |  |  |
| **Continuous** | 0.12 | 0.05 | 4.68 | 0.03 | 1.12 | 1.01 | 1.25 |
| **AF Risk Status at Baseline (ref = Low-risk)** |  |  |  |  |  |  |  |
| **High-risk** | -0.14 | 0.22 | 0.38 | 0.54 | 0.87 | 0.57 | 1.35 |
| **Other Baseline Risk Factors (Yes/No)** |  |  |  |  |  |  |  |
| **Infection** | 0.33 | 0.14 | 5.31 | 0.02 | 1.39 | 1.05 | 1.85 |
| **Hypertension** | 0.06 | 0.15 | 0.17 | 0.68 | 1.07 | 0.79 | 1.44 |
| **Anemia** | 0.65 | 0.15 | 18.68 | <.0001 | 1.91 | 1.42 | 2.56 |
| **Fatigue/asthenia** | 0.23 | 0.15 | 2.30 | 0.13 | 1.27 | 0.93 | 1.71 |
| **Hemorrhage/bleeding** | 0.10 | 0.17 | 0.32 | 0.57 | 1.10 | 0.78 | 1.55 |
| **Atrial fibrillation** | 0.57 | 0.35 | 2.75 | 0.10 | 1.78 | 0.90 | 3.51 |
| **Treatment Regimen** (ref = Rituximab)** |  |  |  |  |  |  |  |
| **R-CHOP** | 0.08 | 0.18 | 0.20 | 0.65 | 1.09 | 0.76 | 1.55 |
| **B-R** | -0.67 | 0.21 | 10.14 | 0.00 | 0.51 | 0.34 | 0.77 |
| **Ibrutinib** | 0.29 | 0.27 | 1.17 | 0.28 | 1.34 | 0.79 | 2.26 |
| *Total number of events is: Neutropenia n=672; secondary malignancy n=196; anemia n=167; infection n=158; thrombocytopenia n=99; atrial fibrillation n=29; hemorrhage/bleeding n=58.  **Number of patients with each regimen (first-line setting) is: rituximab n=467; R-CHOP n=661; B-R n=347; ibrutinib n=112.  Abbreviations: AF, atrial fibrillation; B-R, bendamustine-rituximab; CCI, Charlson comorbidity index; CI, confidence interval; HMO, health maintenance organization; POS, point of service;  PPO, preferred provider organization; R-CHOP, cyclophosphamide/doxorubicin/vincristine/rituximab | | | | | | | |

**Supplemental Table 5. Multivariable Generalized Linear Model Assessing Association Between Incident Adverse Events of Interest and Monthly All-Cause Health Care Costs per Patient During the Entire Study Follow-up**

| **Outcome:  All-Cause Healthcare Costs PPPM** | **Patients Receiving First Observed Treatment for the Four Most Common Regimens** | | | | | | | | |
| --- | --- | --- | --- | --- | --- | --- | --- | --- | --- |
|  | **n = 1,614** | | | | | | | | |
|  | **Parameter Estimate** | **Standard Error** | **Chi-Square** | **p-value** | **95% CI** | | **Cost Ratio** | **95% CI** | |
| **Incident Adverse Event* (Yes/No)** |  |  |  |  |  |  |  |  |  |
| **Neutropenia** | 0.00 | 0.05 | 0.01 | 0.93 | -0.09 | 0.10 | 1.00 | 0.91 | 1.11 |
| **Secondary malignancy** | -0.16 | 0.06 | 5.87 | 0.02 | -0.28 | -0.03 | 0.85 | 0.75 | 0.97 |
| **Anemia** | 0.32 | 0.07 | 18.52 | <.0001 | 0.17 | 0.46 | 1.38 | 1.19 | 1.59 |
| **Infection** | 0.09 | 0.08 | 1.45 | 0.23 | -0.06 | 0.24 | 1.10 | 0.94 | 1.27 |
| **Thrombocytopenia** | 0.50 | 0.09 | 30.55 | <.0001 | 0.32 | 0.67 | 1.64 | 1.38 | 1.96 |
| **Atrial fibrillation** | 0.13 | 0.16 | 0.67 | 0.41 | -0.19 | 0.45 | 1.14 | 0.83 | 1.57 |
| **Hemorrhage/bleeding** | 0.19 | 0.12 | 2.84 | 0.09 | -0.03 | 0.42 | 1.21 | 0.97 | 1.52 |
| **Age at Index** |  |  |  |  |  |  |  |  |  |
| **Continuous** | -0.01 | 0.00 | 11.69 | 0.00 | -0.01 | 0.00 | 0.99 | 0.99 | 1.00 |
| **Region (ref = South)** |  |  |  |  |  |  |  |  |  |
| **Northeast** | 0.12 | 0.06 | 3.95 | 0.05 | 0.00 | 0.23 | 1.13 | 1.00 | 1.26 |
| **Midwest** | 0.12 | 0.05 | 5.15 | 0.02 | 0.02 | 0.22 | 1.13 | 1.02 | 1.25 |
| **West** | 0.13 | 0.07 | 3.06 | 0.08 | -0.02 | 0.27 | 1.14 | 0.98 | 1.32 |
| **Unknown** | -0.32 | 0.24 | 1.71 | 0.19 | -0.79 | 0.16 | 0.73 | 0.45 | 1.17 |
| **Plan Type (ref = PPO)** |  |  |  |  |  |  |  |  |  |
| **HMO** | -0.26 | 0.07 | 14.82 | 0.00 | -0.39 | -0.13 | 0.77 | 0.68 | 0.88 |
| **POS** | 0.11 | 0.09 | 1.43 | 0.23 | -0.07 | 0.29 | 1.12 | 0.93 | 1.34 |
| **Other and Unknown** | 0.21 | 0.12 | 3.43 | 0.06 | -0.01 | 0.44 | 1.24 | 0.99 | 1.55 |
| **CCI Score** |  |  |  |  |  |  |  |  |  |
| **Continuous** | 0.07 | 0.02 | 12.26 | 0.00 | 0.03 | 0.11 | 1.07 | 1.03 | 1.11 |
| **AF Risk Status at Baseline (ref = Low-risk)** |  |  |  |  |  |  |  |  |  |
| **High-risk** | -0.13 | 0.07 | 2.92 | 0.09 | -0.27 | 0.02 | 0.88 | 0.76 | 1.02 |
| **Other Baseline Risk Factors (Yes/No)** |  |  |  |  |  |  |  |  |  |
| **Infection** | -0.04 | 0.05 | 0.76 | 0.38 | -0.13 | 0.05 | 0.96 | 0.88 | 1.05 |
| **Hypertension** | 0.13 | 0.05 | 7.12 | 0.01 | 0.04 | 0.23 | 1.14 | 1.04 | 1.26 |
| **Anemia** | 0.20 | 0.05 | 16.67 | <.0001 | 0.11 | 0.30 | 1.22 | 1.11 | 1.35 |
| **Fatigue/asthenia** | 0.14 | 0.05 | 7.48 | 0.01 | 0.04 | 0.25 | 1.15 | 1.04 | 1.28 |
| **Hemorrhage/bleeding** | -0.02 | 0.06 | 0.13 | 0.72 | -0.14 | 0.10 | 0.98 | 0.87 | 1.10 |
| **Atrial fibrillation** | 0.30 | 0.13 | 5.79 | 0.02 | 0.06 | 0.55 | 1.35 | 1.06 | 1.73 |
| **Treatment Regimen (ref = Rituximab)** |  |  |  |  |  |  |  |  |  |
| **R-CHOP** | 0.09 | 0.06 | 2.53 | 0.11 | -0.02 | 0.20 | 1.09 | 0.98 | 1.22 |
| **B-R** | 0.16 | 0.06 | 7.17 | 0.01 | 0.04 | 0.28 | 1.18 | 1.04 | 1.33 |
| **Ibrutinib** | 0.40 | 0.09 | 19.57 | <.0001 | 0.22 | 0.57 | 1.49 | 1.25 | 1.77 |
| Abbreviations: AF, atrial fibrillation; B-R, bendamustine-rituximab; CCI, Charlson comorbidity index; CI, confidence interval; HMO, health maintenance organization; POS, point of service;  PPO, preferred provider organization; PPPM, per patient per month; R-CHOP, cyclophosphamide/doxorubicin/vincristine/rituximab | | | | | | | | | |
